# AGE-RAGE axis culminates into multiple pathogenic processes: a central road to neurodegeneration

**DOI:** 10.3389/fnmol.2023.1155175

**Published:** 2023-05-17

**Authors:** Reshmee Bhattacharya, Mohammad Rizwan Alam, Mohammad Azhar Kamal, Kyung Jin Seo, Laishram Rajendrakumar Singh

**Affiliations:** ^1^Dr. B. R. Ambedkar Center for Biomedical Research, University of Delhi, Delhi, India; ^2^Department of Hospital Pathology, College of Medicine, Uijeongbu St. Mary’s Hospital, The Catholic University of Korea, Seoul, Republic of Korea; ^3^Department of Pharmaceutics, College of Pharmacy, Prince Sattam Bin Abdulaziz University, Al-kharj, Saudi Arabia

**Keywords:** AGE, RAGE, glycation, neurodegeneration, Velcade

## Abstract

Advanced glycation end-products (AGEs; e.g., glyoxal, methylglyoxal or carboxymethyl-lysine) are heterogenous group of toxic compounds synthesized in the body through both exogenous and endogenous pathways. AGEs are known to covalently modify proteins bringing about loss of functional alteration in the proteins. AGEs also interact with their receptor, receptor for AGE (RAGE) and such interactions influence different biological processes including oxidative stress and apoptosis. Previously, AGE-RAGE axis has long been considered to be the maligning factor for various human diseases including, diabetes, obesity, cardiovascular, aging, etc. Recent developments have revealed the involvement of AGE-RAGE axis in different pathological consequences associated with the onset of neurodegeneration including, disruption of blood brain barrier, neuroinflammation, remodeling of extracellular matrix, dysregulation of polyol pathway and antioxidant enzymes, etc. In the present article, we attempted to describe a new avenue that AGE-RAGE axis culminates to different pathological consequences in brain and therefore, is a central instigating component to several neurodegenerative diseases (NGDs). We also invoke that specific inhibitors of TIR domains of TLR or RAGE receptors are crucial molecules for the therapeutic intervention of NGDs. Clinical perspectives have also been appropriately discussed.

## Introduction

Neurodegenerative diseases (NGDs) including Alzheimer’s disease; AD, Parkinson’s disease; PD, Huntington disease, familial amyloid polyneuropathy (FAP), Amyotrophic lateral sclerosis (ALS), Pick’s disease, etc. are heterogenous group of diseases characterized by the irreversible neuronal damage (Soto and Estrada, 2008). Most of these NGDs are believed to be multifactorial including genetic (mutations) as well as non-genetic (oxidative stress, post-translational modifications, inefficient proteasome activity, mitochondrial dysfunction, etc.) that ultimately results in protein misfolding and development of pathogenic protein inclusions (Barnham et al., 2004; Didonna et al., 2016; Zheng et al., 2016; Wu et al., 2019). Mutations in amyloid-β peptide (Aβ) and its consequent formation of extracellular plaques (Hardy and Allsop, 1991; Agarwal et al., 2020), mutations including duplication and triplication of alpha-synuclein (α-syn), mutation in copper-zinc superoxide dismutase 1 (SOD1) or transthyretin (TTR) result in the formation of amyloids. Furthermore, hyperphosphorylation of Tau also induces neurofibrillary tangles (Iqbal et al., 2010). Abnormality in the processing of amyloid precursor protein by α- and β-secretases also results in the development of Aβ amyloids (Zheng and Koo, 2011). Several other factors including aluminium or pesticide exposures, environmental pollution, traumatic brain injury (Graham and Sharp, 2019), variation in microglial cell density (Muzio et al., 2021), etc. are also major factors contributing to neurodegeneration (Baltazar et al., 2014; Calderón-Garcidueñas et al., 2016; Maya et al., 2016). Chronic bacterial (e.g., *Chlamydia pneumoniae*), a cause of lung infections (Balin et al., 2018); *Borrelia burgdorferi*, the agent of Lyme disease (Gadila et al., 2021); and *Porphyromonas gingivalis* (Dominy et al., 2019), viral (e.g., Human herpesvirus 1, HHV-1 (Duarte et al., 2019); Cytomegalovirus, CMV (Ibekwe and Hanson, 2021); Hepatitis C virus, HCV (Abushouk et al., 2017) and Human herpesvirus 2, HHV-2 (Kristen et al., 2015) or fungal (e.g., *C. glabrata*, *C. famata*, *P. betae*) infections are also potential cause of neurodegeneration (Pisa et al., 2015). In terms of biomarkers, Aβ, tau, α-syn, SOD1 (Hansson, 2021) including certain glycoproteins (Didonna et al., 2016) have also been conventionally used as biomarkers of NGDs. However, Levels of folic acid and polymorphism in rs1051266 does not bear significant association (Mansoori et al., 2014).

Recently, advanced glycation end-products (AGEs) that are populated under various disease pathologies including hyperglycemia, diabetes, obesity, etc. has been implicated in neurodegeneration and therefore, the association of these NGDs. AGEs are heterogenous group of toxic compounds primarily generated when proteins are exposed to reducing sugars (Goldin et. al. 2006; Gkogkolou et. al. 2012; Abushouk et al., 2017; Ibekwe and Hanson, 2021). AGEs are constantly synthesized in the body through both exogenous and endogenous pathways. Exogenous sources of AGEs include dietary consumption of high-carbohydrate containing foods, tea, coffee, beer and foods that are fermented, roasted, baked or fried (Luevano-Contreras and Chapman-Novakofski, 2010). Other factors such as cigarette smoking also influence the amount of AGE formation (Hechtman, 2020). Besides, AGEs are also formed endogenously from reducing sugars (e.g., glucose, fructose, ribose-5-phosphate, etc.) as a by-product of many biological pathways. These reducing sugars are known to covalently modify proteins and enzymes specifically at lysine residues (a process termed as glycation), making them non-functional. Such modification (known as Maillard reaction) is initiated by a nucleophilic addition between a free amino group of protein and electrophilic carbonyl groups of glucose forming an unstable Schiff’s base (Aldimine). Subsequently, the Schiff’s base undergoes Amadori rearrangement to form a stable ketoamine called Amadori product. The Amadori products further undergo enolization reactions to form various dicarbonyl compounds, which upon oxidative and non-oxidative reactions yield AGEs (Sharma et al., 2021). These AGE species include glyoxal, GO; methylglyoxal, MGO; N-ε-Carboxymethyl-lysine, CML; N-ε-carboxyethyl-lysine, CEL etc. AGEs are known to covalently modify proteins much faster than the precursor, reducing sugars (around 20,000-fold more reactive) bringing about functional alteration (Mostafa et al., 2007). AGEs have also been known to interact with receptor for AGEs (RAGEs) resulting in the dysregulation of cell signaling and inflammatory response (Kierdorf and Fritz, 2013). In recent decades, the involvement of such AGE-RAGE axis in various NGDs has been an intellectual curiosity to several researchers. Furthermore, many molecular insights of their role in the pathogenesis of various NGDs have also been documented. In the present article, we attempted to explore a novel avenue that AGE–RAGE axis amounts to multiple pathological consequences and therefore is a central route to neurodegeneration. We also propose that AGE–RAGE axis might culminate to different disease states related to brain.

### Elevated AGE levels is associated with neurodegenerative disorders

In general, high levels of AGEs are found in patients with NGDs. For instance, elevated levels of CML and pentosidine were found in cerebrospinal fluid of AD patients (Bär et al., 2003). AGE-derived products like glyceraldehyde and MGO are also known to accumulate in the brain (Butterfield et al., 2010). Glyceraldehyde occurs in the cytosol of neurons in the hippocampus and para-hippocampal gyrus and generates dicarbonyls by inhibiting the catalytic activity of glyceraldehyde-3-phosphate dehydrogenase. AGE in Creutzfeldt–Jakob disease (CJD) was recently detected in the occipital lobe of patients using anti-AGE and anti-RAGE antibodies. It was revealed that prion protein, PrP-positive granules colocalize with AGEs and AGE-receptors in the astrocyte of patients with CJD (Sasaki et al., 2002). High levels of CML are also observed in cortical neurons and blood vessels of older adults (Yaffe et al., 2011). In the case of ALS, AGEs specifically CEL, CML, pyrraline were up-regulated but are confined to the spinal cord astrocytes (Shibata et al., 2002). Presence of MGO and MGO-derived AGE compounds in the serum of FAP patients have also been reported (Yaffe et al., 2011). High levels of CML and CEL were detected in the serum of multiple sclerosis patients with CML levels being significantly elevated (Damasiewicz-Bodzek et al., 2020). Additionally, methylglyoxal-derived hydroimidazolone (MGO-H1) was also found to be upregulated in patients with multiple sclerosis lesions (Wetzels et al., 2019). Histochemical analysis of PD patients also revealed that AGE and RAGE levels were increased in frontal cortex relative to controls (Juranek et al., 2015). Studies have also shown that there is a positive correlation between the peripheral AGE levels and cognitive decline in older adults with and without diabetes (Dyer et al., 2020). MGO is also reported to play an important role in cognitive decline and neurodegeneration in older people. Thus, different AGEs are upregulated under various NGDs *via* specifically contributing to the cognitive impairment.

### Lowered level of de-glycating enzymes is linked with neurodegeneration

Cells or tissues have evolved enzymatic mechanisms to detoxify AGEs with the help of various enzymes. Glyoxalases (GLO), comprising of GLO-I, II and III, are involved in the detoxification of reactive α-oxoaldehydes to D-lactate and oxalate (Distler and Palmer, 2012). MGO is detoxified by the glyoxalase system into D-lactate with GLO-1 as the key enzyme (Allaman et al., 2015). Additionally, AGEs are also reported to be detoxified by an enzyme called Fructosamine 3-kinase (FN3K). FN3K is known to phosphorylate and destabilize amadori products leading to their spontaneous breakdown (van Schaftingen et al., 2012). It has been shown that patients in early stages of AD have upregulated levels of GLO1 but gradually downregulates in the middle and late stages of AD (Kuhla et al., 2007). Insufficient function of the glyoxalase system has also been known to increase GSSG/GSH ratio in patients with AD, which can further aggravate disease severity (Maessen et al., 2015). Additionally, a decrease in GLO-1 activity, accompanied by increased carbonyl stress has been reported in some PD, schizophrenic and familial sporadic cases of ALS patients (Arai et. al. 2010; Toyosima et al. 2011; Distler and Palmer, 2012; Dyer et al., 2020).

### Protein covalent modification by AGEs results in functional impairment and formation of toxic protein inclusions associated with various NGDs

**Figure 1:**
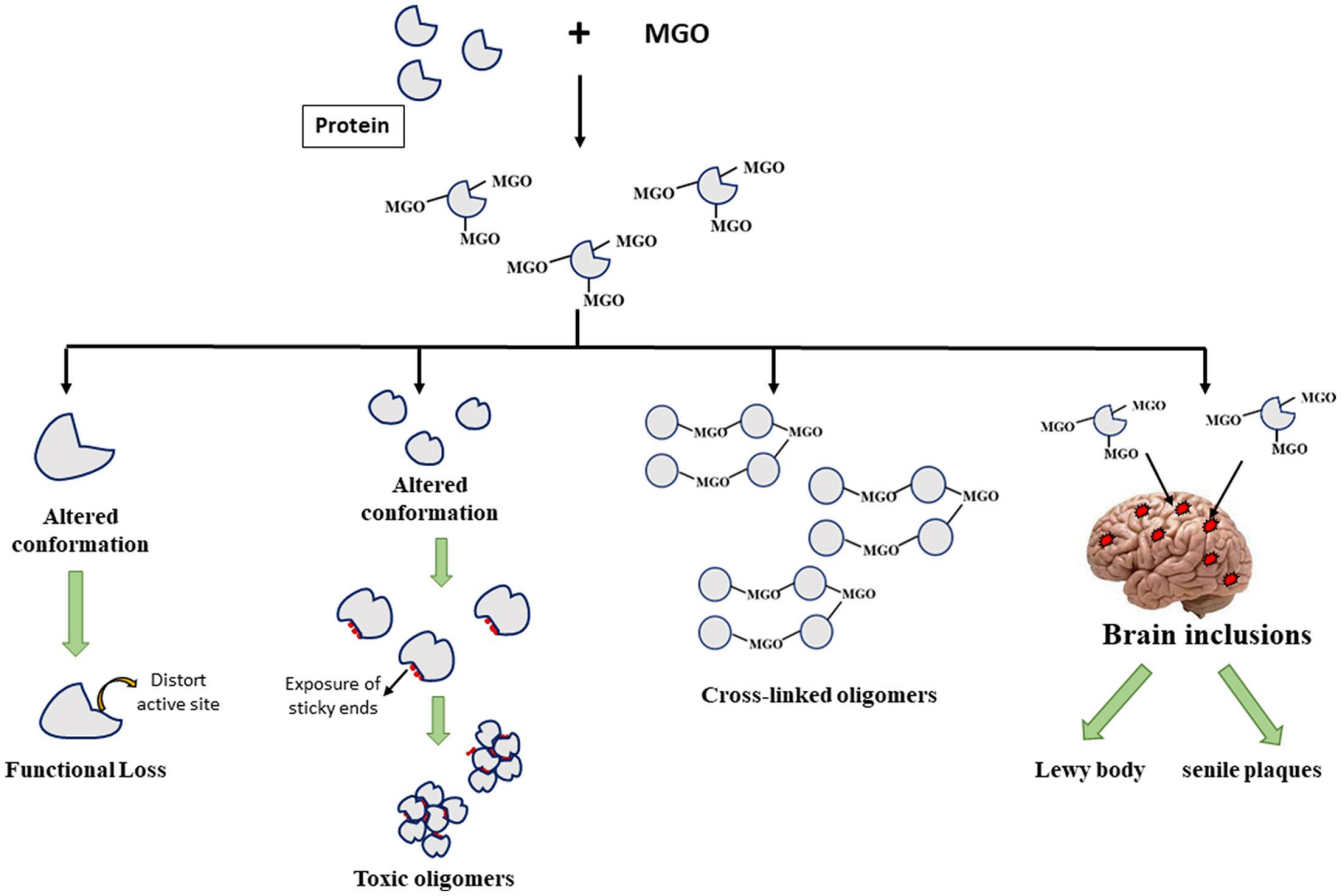
Fates of Advanced glycation end-product (AGE)-induced glycation of different proteins.

It has been understood that post-translational modification of proteins brings about alteration in the native state structural integrity leading to at least 2 consequences (Figure 1). The structural change could influence the integrity of the active pocket making the enzyme deficient in picking substrates properly. It may also lead to exposition of hydrophobic clusters (that were otherwise buried to the core) making the protein aggregation prone species that ultimately form toxic oligomers. Since, AGEs are dicarbonyls, formation of a covalent adduct with one lysine residue on a polypeptide chain generates a free carbonyl group which can further form another covalent adduct with the lysine residues on the other polypeptides developing a cross-linked oligomer. As a result, there is toxic gain in function of the modified proteins. Furthermore, as described in the previous section, certain protein inclusions, e.g., LB (in case of PD) and senile plaques (in case of AD) which are the molecular hallmarks of various NGDs contain such glycated proteins or AGEs that are believed to aggravate their cytotoxicity. Thus, glycated proteins contribute to the formation of different toxic protein inclusions and their neurotoxicity.

Table 1 summarizes a list of important proteins in the brain along with their glycation sites and the nature of toxic inclusions formed. It is seen in this table that almost all these proteins are the major constituents of the protein inclusions associated with the respective NGDs. For instance, plaque fractions of AD patients contain high levels of AGEs and glycated Aβ proteins as compared to the control groups (Vitek et al., 1994). Furthermore, immunohistochemical studies have demonstrated that brains of patients with both hyperglycemia and AD complications have higher levels of glycated-Aβ and Tau positive cells inducing neurotoxicity *via* upregulation of RAGE and glycogen-synthase kinase-3 (GSK-3) activity (Li et al., 2013).

**Table 1:** List of brain proteins with glycation sites and the nature of toxic inclusions formed.

S. No.	Name of protein	Glycation sites	Nature of toxic inclusions	Disease	References
1	Amyloid-β	K16, R5	Senile plaques	Alzheimer Disease	Tang et al. (2007)
2	Tau	K280, K281, K340, K369, K395	Neurofibrillary tangles	Alzheimer Disease	Mistry et al. (2015)
3	α-Synuclein	K75, K79, K92, K120	Lewy bodies	Parkinson’s Disease	Field-Smith et al. (2006)
4	(SOD 1)	K3, K9, K30, K36, K122 and K128	Amyloid fibril	Amylotropic lateral sclerosis	Qu et al. (2010)
5	TTR	ND	Amyloid fibril	Familial amyloid polyneuropathy	–

ND, not detected.

Brains of PD patients, on the other hand, typically consist of Lewy bodies (LB) located in neurons of substantia nigra. These LB primarily composed of aggregated fragments of the protein, α-syn. Glycation of α-syn is believed to be an important factor that exacerbate neurotoxicity in PD patients (Padmaraju et al., 2011). A review by Guerrero et al. (2013) suggested the possible role of glycated α-syn in inducing toxicity in dopaminergic neurons (Guerrero et al., 2013). They argued that glycation of α-syn results in the development of small oligomers, which are far more cytotoxic than the mature α-syn aggregates. The oligomers as well as their glycated monomeric species have the ability to generate reactive oxygen species (ROS) in neuronal cells and induce oxidative stress. Other studies have also shown that glycated α-syn could bind to DNA and change its conformation from B to a mixture of B-C-A form (Padmaraju et al., 2011). Furthermore, α-syn has been reported to undergo rapid glycation in the presence of D-ribose generating molten globule-like aggregations that cause oxidative stress (Chen et al., 2010).

The most common cause of ALS is believed to be due to missense mutations in SOD1 resulting in the disruption of redox homeostasis. In an important study, it has been revealed that protein glycation is enhanced in ALS patients with SOD1 A4V mutation and in transgenic mice with SOD1 G93A mutant (Shibata et al., 2002). Glycation specifically occurs at lys122 and lys128 and consequently results in lowered enzyme activity and increased oxidative stress. Other studies also indicated that glycation of wild type or mutant SOD1 results in the enzyme inactivation and formation of amyloids (Iannuzzi et al., 2014; Sirangelo et al., 2016).

Defects in TTR protein (both genetic and non-genetic) are known to result in the development of FAP. FAP is an autosomal dominant disease characterized by deposits of TTR amyloid fibrils in various parts of the brain. Studies have unveiled that glycation of TTR may contribute to cellular stress and toxicity in FAP patients by activating NF-κB (Sousa et al., 2001). In another study nerve biopsy samples (containing glycated TTR) of FAP patients showed RAGE-dependent expression of pro-inflammatory cytokines and inducible form of nitric oxide synthase at early stages of the disease, followed by activation of caspase-3 and DNA fragmentation (Sousa et al., 2001).

### Advanced glycation end-products disrupt blood brain barrier

A unique set of blood vessels that are involved in vascularizing the CNS, forms a crucial part of blood brain barrier (BBB; Kadry et al., 2020). BBB mainly consist of endothelial cells, astrocytes and pericytes (Xu et al., 2019). Astrocytes and pericytes wrap around endothelial cells and provide biochemical support. Pericytes adhere to the basement membrane with the help of integrin α. Recent studies have revealed that high level of AGEs (as in case of hyperglycemia) decreases integrin α1 protein levels (Rom et al., 2020). There was also a significant reduction in mRNA levels of PDGF-R1β (Platelet derived growth factor receptor-1β) and Connexin-43 (Cx-43) which are important for pericyte function (Rom et al., 2020). Such decrease in the expression levels was associated with defects in the development of cell polarity and BBB integrity. Furthermore, AGEs have been known to influence the degree of autocrine TGF-β signaling by pericytes resulting in the upregulation of vascular endothelial growth factor and matrix metalloproteinase-2 (MMP-2) leading to basement membrane hypertrophy of the BBB. Increase autocrine signaling also increases of the amount of fibronectin (a protein rich in extracellular matrix, ECM) in the pericytes (Shimizu et al., 2013) indicating injury in the BBB.

It has been well understood that the main purpose of BBB is to allow selective influx of ions, molecules and cells between the blood and brain (Daneman and Prat, 2015). In fact, the selectivity of BBB is mainly due to presence of tight junctions that are present between the endothelial cells of brain capillaries. Tight junctions mainly comprise of proteins like occludins and claudins (Luissint et al., 2012). Occludins play important role in controlling paracellular permeability, stability and optimal barrier function of tight junctions (Yuan et al., 2020). Claudins are also the backbone of tight junctions and help to seal the paracellular space (Krause et al., 2008). Recent studies have identified that the expression of tight junction proteins, occludin and claudin decreases under elevated AGEs that eventually result in losing the integrity of tight junctions (Li et al., 2015). It has also been shown that exposure of brain microvessels to hyperglycemic condition or AGEs *ex vivo* results in the abnormalities in tight junction protein distributions. They also observed that there was large increase in the extracellular vesicles and increased expression of occludin and claudin-5 protein in diabetic mellitus mice. Pericytes exposed to hyperglycemia or AGEs also displayed diminished expression of integrin α1, PDGF-R1β and Cx 43 (AGEs disrupt bold brain barrier). The overall results indicate that hyperglycemic condition is detrimental to BBB physiology. Furthermore, it has also been shown that diabetic patients exhibit macrovascular changes and dysregulation of BBB permeability that further facilitates robust inflammatory response and stroke (BBB dysfunction in ischemic stroke).

### Advanced glycation end-products induce remodeling of extracellular matrix

Extracellular matrix (ECM) is a complex molecular matrix composed of an array of macromolecules comprising of collagen, proteoglycans, and matrix metalloproteinases (MMPs; Lam et al., 2019), etc. It is a highly dynamic structure and undergoes controlled remodeling mediated by specific enzymes called, MMPs (Bonnans et al., 2014). ECM remodeling is important for regulating morphogenesis, and plasticity of neurons. Several lines of evidence suggest that dysregulation of ECM, especially imbalance of MMPs and their inhibitors (TIMPs) is known to be associated with several NGDs (Bonnans et al., 2014). It has been previously shown that presence of high glucose level increases MMP9 activity and oxidative stress with subsequent BBB dysfunction in ischemic rats (Kamada et al., 2007). Similarly, in acute ischemic stroke patients, there was upregulation of AGE level and concomitant increase in MMP9 and oxidative stress (Jayaraj et al., 2019). Furthermore, AGEs have also been reported to stimulate brain aneurysm in mouse models by promoting MMP9 expression (Zhang et al., 2011). In addition to MMPs, AGEs are also found to affect other ECM proteins including laminin and fibronectin in diabetic rat models (Duran-Jimenez et al., 2009). Additionally, glycation of collagen proteins also results in slow turnover due to crosslinking of collagen and hence increasing the stiffness of the ECM matrix (Furber, 2006). Deposition of glycated toxic proteins including, Aβ, α-syn or TTR has also been known to induce remodeling of ECMs by affecting expression level of various MMPs (Cao et al., 2013; Fournet et al., 2018; Valdinocci et al., 2018). Thus, AGEs and glycated proteins bring about alteration of the brain ECM and consequently results in pathological consequences.

### Dysregulation of AGE-RAGE axis causes neuroinflammation

**Figure 2:**
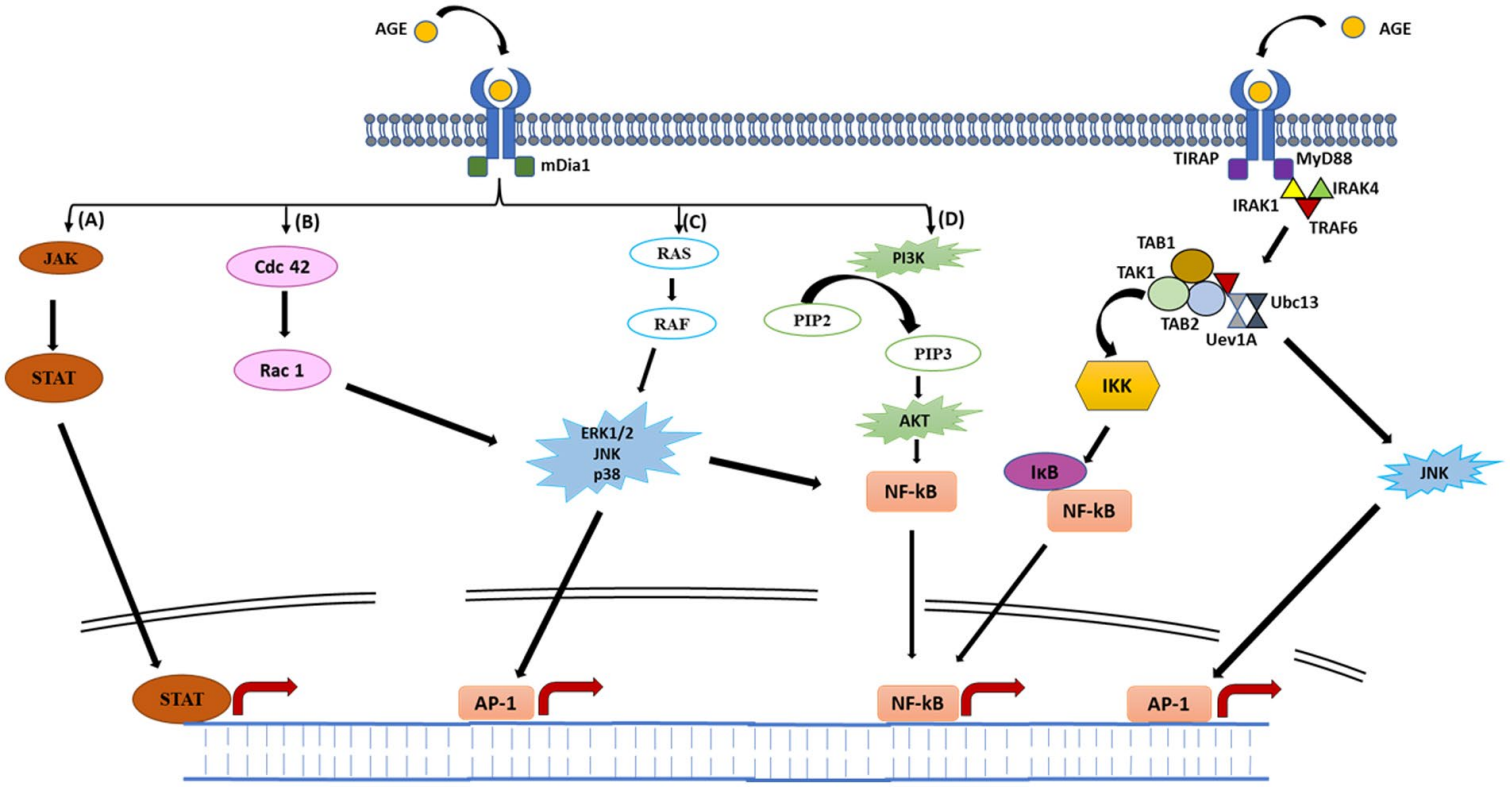
Schematic representation of AGE–RAGE signaling pathways. Binding of AGE to RAGE results in its dimerization followed by recruitment of adaptor protein (mDia1) to the cytoplasmic domain of RAGE. Recruitment of mDia1 activates four major signaling pathways. **(A)**, mDia1 recruits JAK which then phosphorylates STAT to form dimers. Activated STAT then translocate to the nucleus, enabling expression of various cytokine-responsive genes, leading to inflammatory response. **(B)** Binding of mDia1 may stimulate switching of GDP-bound cdc 42 to its active GTP-bound state. Activated cdc42 further activates Rac-1 which phosphorylates downstream MAPKs (JNK and p38). Phosphorylation of JNK and p38 results in the activation of AP-1 and NF-κB transcription factors. **(C)** mDia1 again may recruit SOS, wherein it activates RAS by exchanging GDP to GTP. GTP-bound RAS further activates RAF finally initiating phosphorylation cascade by activating MAPKs (ERK1/2, p38 and JNK). These MAPKs translocate to the nucleus and activates AP-1 transcription factor. ERK/p38/JNK may also activate NF-κB in the cytoplasm, regulating various genes involved in inflammatory response. **(D)** mDia1 may also stimulate activation of PI3K protein. Activated PI3K further phosphorylates PIP2 to form PIP3, which in turn phosphorylates and activates AKT. Once activated, AKT further stimulate NF-κB which moves to the nucleus and regulates transcription of several genes. Activated RAGE may also bind to other adaptor proteins (MyD88 and TIRAP) initiating a phosphorylation event ultimately leading to NF-κB activation (see text for detail explanation).

**Figure 3:**
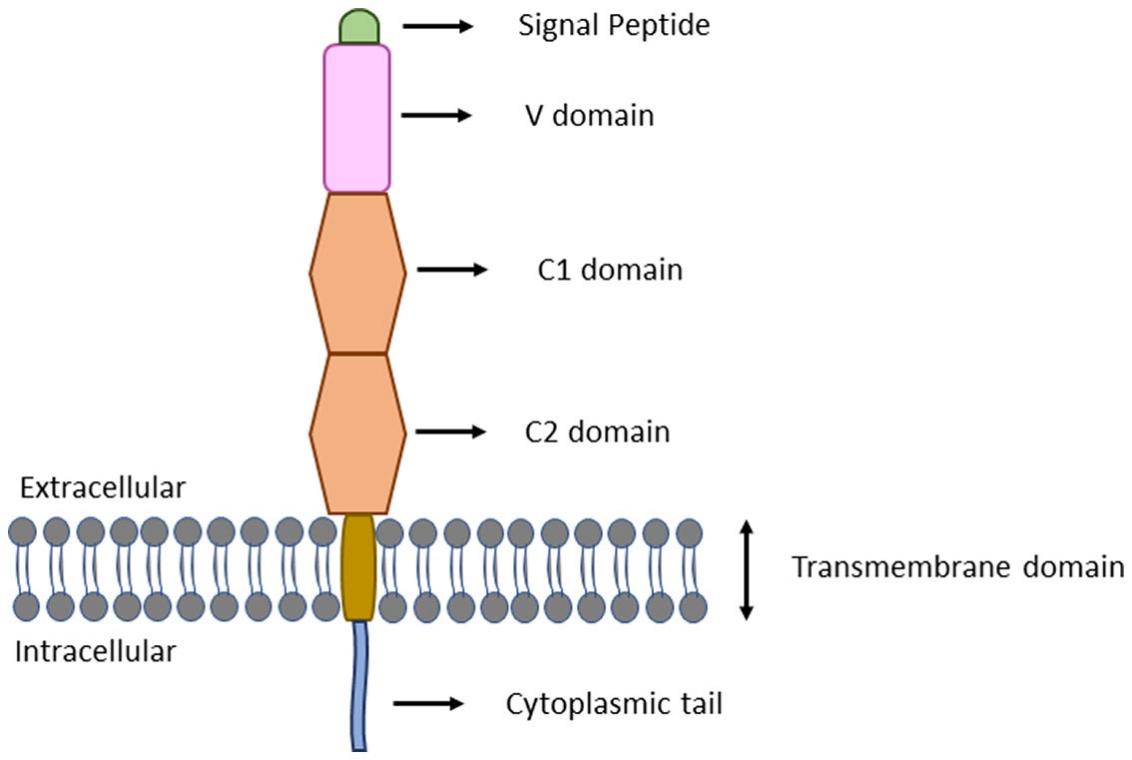
Structure of full-length RAGE. RAGE consists of three extracellular domains (V, C1, and C2), a transmembrane region and an intracellular cytoplasmic tail. The V-domain contains 23–116 amino acid residues and functions as ligand binding site. The C1 and C2 domains contain 124–221 and 227–317 amino acid residues, respectively. The transmembrane spinning helix consists of 343–363 amino acid residues. The short cytoplasmic tail containing 364–404 amino acid residues functions as signal transduction initiator.

The AGE-RAGE interaction is a potential mechanism underlying the AGE-induced inflammatory responses. RAGE is a 35 kDa multi-ligand transmembrane receptor that belongs to the immunoglobulin gene superfamily (Krause et al., 2008; Li et al., 2015). Similar to toll-like receptors (TLRs), RAGEs are also considered to be pattern recognition receptors predominantly believed to be involved in recognition of endogenous molecules released during physiological stress or chronic inflammation. RAGE is expressed in a myriad of immune cell types, including neutrophils, monocytes-macrophages, lymphocytes, antigen presenting cells, as well as regulatory T cells (Li et al., 2015; Lam et al., 2019). Interaction of RAGE with AGE occurs mainly through electrostatic interactions between positive charges present on V and C1 domains of RAGE (Figure 2) and negative charges present on ligands (Koch et al., 2010). On exposure of RAGE to its ligands, first of all RAGE undergoes dimerization which then recruits adaptor protein (mDia1) by the cytoplasmic domain of RAGE (Derk et al., 2018; Figure 3). The RAGE-mDia1 signaling is important for the activation of at least 4 independent pathways (Figure 3). It is seen in this figure that mDia1 activates MAPKs including ERK1/2, p38 and c-JNK leading to the activation of their respective substrate, transcription factors which eventually enters the nucleus and transcribes downstream genes responsible for cell proliferation and apoptosis. The adaptor protein, mDia1 also activates other signaling pathways including JAK/STAT, Ras/Rac, Cdc42 and PI3K/Akt (Ramasamy et al., 2005; Hudson et al., 2008). Activated RAGE also binds to the other adaptor proteins involved in the innate immunity including MyD88 and TIRAP (Sakaguchi et al., 2011). MyD88/TIRAP in turn recruits IRAK4/IRAK1/TRAF6. Phosphorylation of IRAK1 by IRAK4 results in the release of TRAF6 from the receptor complex. TRAF6 further forms complex with TAK1/TAB1/TAB2 protein complex and additionally with Ubc13 and Uev1 proteins. The activated TAK1 phosphorylates IKK proteins leading to the phosphorylation of IKB that associates with NF-κB. The phosphorylated IKB becomes ubiquitinylated and therefore, can no longer hold NF-κB to the cytosol. The released NF-κB translocates to the nucleus and initiates transcription of genes responsible for inflammation and apoptosis. TAK1 may also phosphorylate JNK (an MAPK) and further activate AP1 transcription factors (Akira, 2003).

RAGEs have also been reported to bind to non-AGE ligands. Aβ and many other protein aggregates are known to be a ligand for RAGE (Bongarzone et al., 2017). Interaction of RAGE with Aβ peptides on the surface of neuronal cells elicits NF-κB-mediated secretion of macrophage-colony stimulating factor (M-CSF) and pro-inflammatory cytokines. M-CSF further interacts with RAGE resulting in chemotaxis, cell proliferation and increased oxidative stress etc. Other non-AGE ligand for RAGE includes lipopolysaccharide, LPS; S100 family, complement component protein, C3a; High-mobility group box 1 protein, HMGB1; phosphatidylserine, PS; macrophage 1 antigen (Mac-1/CD11b) etc. and are involved in inflammatory responses (Krause et al., 2008; Jayaraj et al., 2019). These non-AGE ligands are often dysregulated under hyperglycemic conditions (Kosaki et al., 2004; Lee and Park, 2013; Manigrasso et al., 2014) and therefore, affects normal neuronal physiology. RAGE interaction with PECAM-1 facilitates the migration of circulating monocytes across the blood brain barrier thereby inducing BBB dysfunction because of inflammatory response. Mac-1, a member of the β2-integrin family, helps in adhesion of leukocytes to endothelial cells of the brain. Interaction between Mac-1 and RAGE has been also described as a potential factor for the recruitment of leukocytes in inflammatory responses (Orlova et al., 2007). Upregulation and release of S100 to the extracellular matrix could also initiate interaction with RAGE that eventually activates NF-κB, inducing the production of pro-inflammatory cytokines and migration of neutrophils, monocytes, and macrophages (Xia et al., 2018). Additionally, RAGE and TLR4 play an important role in the inflammatory response against HMGB1, a late pro-inflammatory cytokine. HMGB1 binds to RAGE, leading to mitogen-activated protein kinase (MAPK) activation, thereby promoting TLR4 translocation on the cell surface (Zhong et al., 2020). HMGB1 has also been reported to activate dendritic cells (DCs) and helps in cell proliferation and migration.

### Activation of AGE–RAGE axis induces oxidative stress and apoptosis

Increased oxidative stress is a major hallmark of NGDs. As explained above, engagement of AGE with RAGE generates inflammatory response *via* activation of cdc42 that ultimately upregulates Rac1. Indeed, it is Rac1 that is responsible for the binding of the cytosolic NADPH oxidase (NOX) subunit to its membrane-bound catalytic subunit (Raz et al., 2010). Thus, the Rac1-dependent inflammatory pathway is also linked with the activation of NOX and hence the production of reactive oxygen species (ROS). In another development, RAGE engagement by AGEs also induces a hyper-responsive cellular state in macrophage and monocytes, resulting in an increased secretion of pro-inflammatory cytokines, including IL-12, insulin-like growth factor, TNF-α, etc. (Tomino et al., 2011; Jin et al., 2015). These events further helped to increase the production of ROS and reactive nitrogen intermediates. AGE has also been reported to inhibit nitric oxide synthase activity leading to the upregulation of ROS (Chuah et al., 2013; Iannuzzi et al., 2014; Sirangelo et al., 2016). Additionally, AGEs are products of both oxidative and non-oxidative processes. The precursor molecules (reducing sugars) can undergo oxidation in presence of oxygen and transition metal ions generating H_2_O_2_ and other oxygen radicals. In a systematic study, glycation of amino acids with MGO were found to involve generation of three types of radicals (Yim et al., 1995). These radicals are a cross-linked radical cation (the methylglyoxal dialkylimine radical cation or its protonated cation), the methylglyoxal radical anion, and the superoxide radical anion (which are formed only in the presence of oxygen molecules). The generation of the cross-linked radical cation and the methylglyoxal radical anion does not require metal ions or oxygens, suggesting that they are formed by a direct 1-electron transfer process. The radical species thus generated, may further initiate modification of different proteins *via* free radical chain reactions. Such a kind of reaction in presence of glycated proteins may increase peroxidation of lipids which therefore, contribute to accelerating oxidative modification of vascular walls.

One of the potential mechanisms of AGE-induced deleterious effects is *via* programmed cell death (Mahali et al., 2011). AGEs have been reported to induce apoptosis in various cultured cells including microvascular cells, neuronal cells, fibroblasts, and renal mesangial cells (Alikhani et al., 2007). As mentioned in the previous section, AGEs have been shown to activate serine–threonine protein kinases in the MAPK-dependent pathways. Similarly, AGEs are known to induce osteoblast cells apoptosis *via* both JNK and p38 MAPK (Alikhani et al., n.d.). It has also been known that AGE-RAGE interaction alters the expression of many cellular proteins, which might affect cellular processes such as apoptosis (Mahali et al., 2011). Furthermore, such AGE-RAGE interaction causes release of vesicular contents, including interleukin-8 (IL-8). The degranulated IL-8 binds to its receptors, IL-8Rs, and causes sequential events in cells, including an increase in intracellular Ca^2+^, activation of calcineurin, and dephosphorylation of cytoplasmic NF-AT (Nuclear factor of activated T cells). NF-AT then translocates to the nucleus and induces expression of FasL (Mahali et al., 2011). Elevated FasL increases activity of caspases and induces cell death. In another study high levels of MGO have shown to increase activity of the pro-apoptotic protein, Bax and reduces the anti-apoptotic protein Bcl-2 expression in neuroblastoma cells (Guo et al., 2016; Lee et al., 2020) and even disrupts mitochondrial integrity (Lai et al., 2020).

### Advanced glycation end-product impairs anti-oxidant enzymes of brain

One important aspect of neurodegeneration is the decrease efficiency of antioxidant system leading to lowered activity to reduce ROS. It has been reported that increase accumulation of AGEs also results in the decrease expression of various anti-oxidant enzyme systems. For instance, activity of catalase, SOD1 and glutathione peroxidase (GPx) has been shown to be decreased in the plasma of familial and sporadic ALS patients (Nikolić-Kokić et al., 2006). GPx has also been observed to be inactivated by glyceraldehyde, MGO and 3-DG (Niwa and Tsukushi, 2001). A correlation between AGEs and total antioxidant system has also been revealed in peripheral artery diseases (Lapolla et al., 2007). Specifically, it was observed that there was upregulation of pentosidine and a concomitant decrease in the anti-oxidant systems including vitamin E (Lapolla et al., 2007). Levels of glutathione peroxidase and SOD were also found to be diminished in *in vitro* diabetic encephalopathy model, HT22 cells (Gan et al., 2021).

### Advanced glycation end-product activates polyol pathway and induces neuropathy

The polyol pathway converts sugars such as glucose into sugar alcohols (polyols). For example, glucose is converted into sorbitol by the action of enzyme aldose reductase (Steele et al., 2008). Under normal conditions glucose is metabolized *via* hexokinase pathway to generate pyruvate. However, in hyperglycemic patients, high glucose levels saturate the hexokinase pathway and glucose is then metabolized *via* polyol pathway in a two-step process (Anusha Chauhan et al. n.d.). Accordingly, glucose is first reduced to sorbitol with the help of aldose reductase, which is then oxidized to fructose with the help of sorbitol dehydrogenase. Furthermore, this polyol pathway has a domino effect on other metabolic processes. For instance, increased polyol pathway activity alters the redox state of the pyridine nucleotides NADP + and NAD +, thereby reducing their concentrations (Tsioufis et al., 2018). Several studies have evidenced that the polyol pathway is activated under elevated AGE conditions including diabetes, hyperglycemia, obesity, etc. Increased aldose reductase activity and accumulation of sorbitol have also been found in diabetic animal models (Iwata et al., 2007; Lim et al., 2010).

Increased flux of polyols as a consequence of persistent hyperglycemic (or elevated AGE levels) conditions is also believed to be one of the basic causes of peripheral nerve damage (Yagihashi et al., 2011). Such condition arising as a result of prolonged hyperglycemia is commonly referred to as diabetic neuropathy. Previous reports suggested accumulation of high sorbitol and fructose levels within the nerve endings of diabetic neuropathic patients (Akamine et al., 2018). High levels of sorbitol and fructose caused by the activation of aldose reductase and sorbitol dehydrogenase inhibition were also observed in sciatic nerve fibers of streptozotocin-diabetic rats (PubMed, 2001). Furthermore, studies conducted using aldose-reductase knockout mice demonstrated that deficiency of the enzyme could prevent diabetes-induced oxidative stress in nerve cells in the retina (Cheung et al., 2005). Hyperactivation of aldose reductase in renal cells have also been reported to be linked with aberrant activation of protein kinase C (Ishii et al., 1998; Kapor-Drezgic et al., 1999; Noh and King, 2007), generation of AGEs, increased expression of TGF-β and generation of ROS (Noh and King, 2007) that might eventually induce apoptotic death of cells. Similarly, deficiency of aldose reductase resulted in reduced oxidative stress in the peripheral nerves of diabetic mice (Ho et al., 2006). All these evidences indicate that enhanced activation of polyol pathway under hyperglycemia contributes to the onset and progression of diabetic neuropathy (Tang et al., 2007).

### Summary and future perspectives

**Figure 4:**
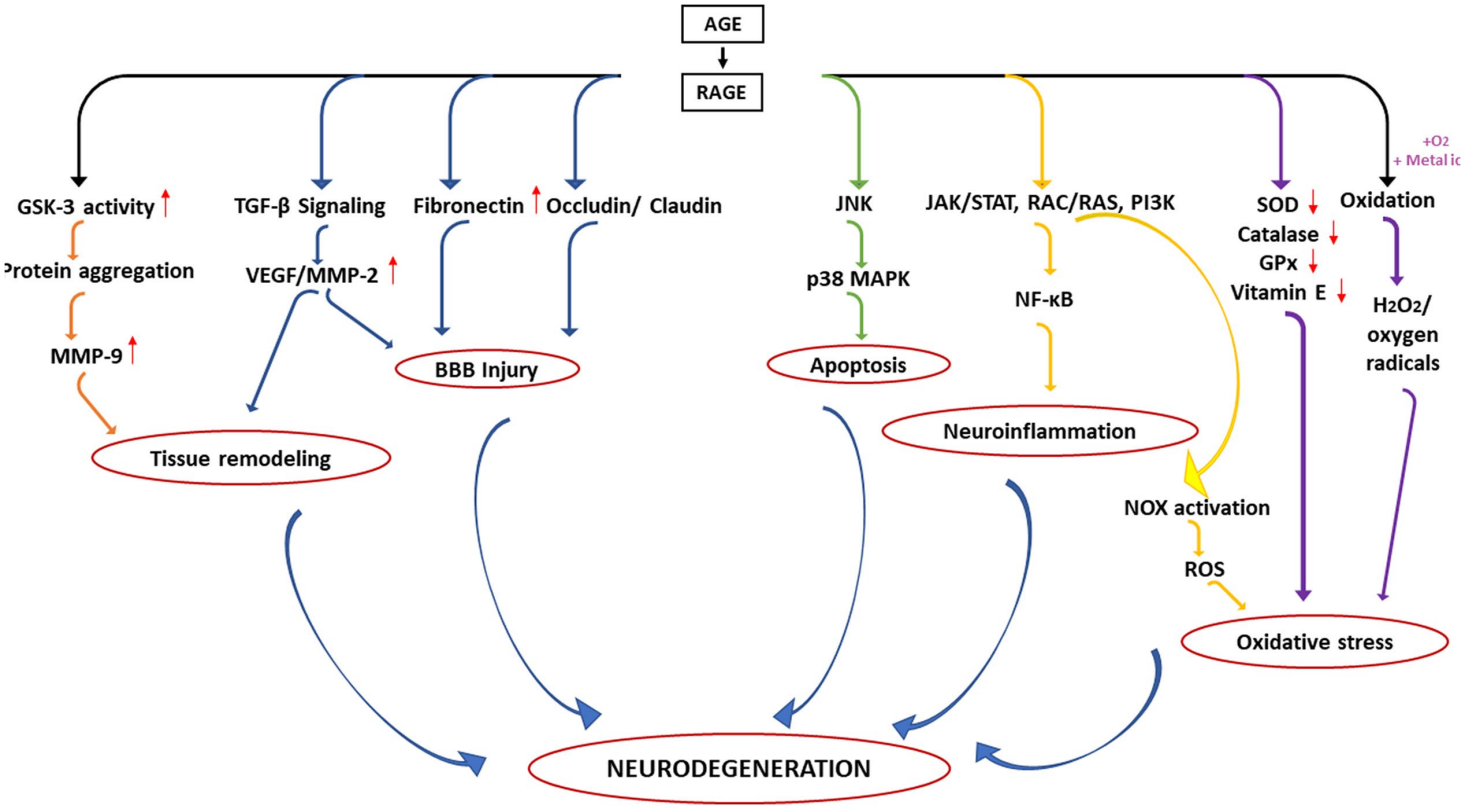
Different pathological consequences of altered AGE–RAGE axis in the neurodegeneration.

It is understood that AGE-RAGE axis becomes a central road toward the development of different pathological consequences associated with NGDs. A summary of the different consequences is depicted in Figure 4. In the first aspect, activation of RAGE results in increased GSK-3 activity thereby enhancing deposition of toxic protein aggregates in the ECM matrix which in turn leads to increase expression of MMP9. On the other hand, activation of RAGE may also influence TGF-β signalling which again activates VEGF/MMP-2. Such upregulation of MMPs and VEGF is primarily responsible for remodeling of the ECM matrix. In the second case, hyperactivation of RAGE might also influence the expression of other proteins associated with the tight junction of endothelial cells surrounding the BBB including fibronectin and claudin/occludin leading to the disruption of BBB mechanics.

Additionally, upregulation of VEGF and MMPs may further augment such BBB injury. AGE-RAGE interaction has also been known to induce apoptosis (third pathway) and neuroinflammation (fourth pathway) *via* upregulation of p38 MAPK and activation of NF-κB, respectively. Upregulation of RAGE also induces oxidative stress (an important hallmark of neurodegeneration) *via* activation of NOX (fifth pathway) and downregulation of several antioxidant enzymes (sixth pathway). AGE may also undergo oxidation in presence of oxygen or transition metals generating free radicals (seventh pathway).

Thus, dysregulation of AGE-RAGE axis results in different aberrant biological/physiological processes leading to neurodegeneration. The observations highlight that RAGE inhibitors should be a potential therapeutic for many NGDs. Several small molecule inhibitors that can specifically bind to the BB loop of the TIR domain of TLR/RAGE have already been developed that are currently in various stages of clinical trial (see Table 2) but none has been licensed for use of NGDs (Mistry et al., 2015). Furthermore, as depicted in Figure 4, the primary cause of the neuronal death is because of chronic inflammatory response initiated due to release of NF-κB. Therefore, potential inhibitors of innate immune inflammatory response specifically targeted at inhibition of NF-κB would be another additional strategy. One potential inhibitor of NF-κB is a proteosome inhibitor, velcade^®^ (bortezomib). Currently, velcade^®^ is used for the treatment of multiple myeloma because proteosome inhibition leads to increase level of IKB resulting (by preventing degradation) in the inhibition of NF-κB (Field-Smith et al., 2006). Since velcade^®^ is an FDA approved drug, an insight to repurpose this molecule against NGDs would be a viable approach. In support of this, there are studies that demonstrated that velcade^®^ is an effective neuroprotective agent for the treatment of traumatic brain injury (Qu et al., 2010). Another study also showed that velcade^®^ therapy protected from cognitive impairment in mice and patients (Huehnchen and Boehmerle, 2020) and is safe medication for central nervous system. However, there are reports that the CNS uptake of velcade^®^ is little poor as the BBB does not allow large molecule to pass through (Huehnchen et al., 2020). Research on this aspect to overcome diffusibility of velcade^®^ across BBB should therefore, be undertaken. Other NF-κB inhibitors such as kyprolis^®^ (cafilzomib) and ninlaro^®^ (ixazomib; used for the treatment of multiple myeloma) should also be investigated for possible therapeutic intervention.

**Table 2:** Potential RAGE inhibitors tested for their ability to treat neurotoxicity.

S. No.	Name	Uses	References
1	Papaverine	Reduces chronic inflammation	Ishii et al. (1998)
2	Tranilast	Inhibits binding interactions of RAGE with S100A11 and S100A12	Ishii et al. (1998)
3	FPS-ZM1	Alzheimer’s disease Parkinson’s disease	Kapor-Drezgic et al. (1999), Noh and King (2007)
4	PF-04494700 (Azeliragon)	Reduces Aβ_1-42_ plaque deposition, Dementia	Ishii et al. (1998); Noh and King (2007)
5	pioglitazone	Epilepsy	Ishii et al. (1998)
6	Gliclazide	Reduces chronic inflammation, Parkinson’s disease	Ishii et al. (1998)
7	F-18 analog of FMS-ZM1	Alzheimer’s disease	Ishii et al. (1998)
8	Emitine	Blocks Aβ_1-42_ and RAGE interaction	Ishii et al. (1998)

## Author contributions

LS conceived the idea. LS and RB wrote the manuscript. LS, MA, MK, and KS reviewed the manuscript. All authors contributed to the article and approved the submitted version.

## Funding

This review is partly supported by IoE, University of Delhi 409 (No./IoE/2021/12/FRP) grant.

## Conflict of interest

The authors declare that the research was conducted in the absence of any commercial or financial relationships that could be construed as a potential conflict of interest.

## Publisher’s note

All claims expressed in this article are solely those of the authors and do not necessarily represent those of their affiliated organizations, or those of the publisher, the editors and the reviewers. Any product that may be evaluated in this article, or claim that may be made by its manufacturer, is not guaranteed or endorsed by the publisher.
